# Phase, Chemical, Thermal, and Morphological Analyses of Thermoplastic Polyurethane (TPU) Nanocomposites Reinforced with Jute Cellulose Nanofibers (CNFs)

**DOI:** 10.3390/polym17070899

**Published:** 2025-03-27

**Authors:** Siti Syazwani Nordi, Ervina Efzan Mhd Noor, Chee Kuang Kok, Nurhidayatullaili Muhd Julkapli, Mirza Farrukh Baig

**Affiliations:** 1 Centre for Manufacturing and Environmental Sustainability (CMES), Faculty of Engineering and Technology, Multimedia University, Ayer Keroh, Melaka 75450, Malaysia; sitisyazwani28@gmail.com (S.S.N.); ckkok@mmu.edu.my (C.K.K.); farrukhbaig@mmu.edu.my (M.F.B.); 2 Nanotechnology and Catalysis Research Center (NANOCAT), Level 3, Block A, Institute for Advanced Studies, University Malaysa, Kuala Lumpur 50603, Malaysia; nurhidayatullaili@um.edu.my

**Keywords:** polymer, nanocomposite, thermoplastic, fiber, bioresources

## Abstract

In response to the growing demand for high-performance materials in industries such as automotive, aerospace, and construction, this study investigates the impact of jute cellulose nanofibers (CNFs) on the chemical, thermal, and morphological properties of thermoplastic polyurethane (TPU) nanocomposites. Jute CNFs were extracted using a chemo-mechanical method and incorporated into TPU through melt blending. Fourier transform infrared (FTIR) spectroscopy revealed notable changes in the chemical structure of the nanocomposites, including intensified O-H stretching vibrations and reduced C-H stretching vibrations upon the addition of 2 wt% and 4 wt% jute CNFs. Strong interfacial interactions between the jute CNFs and the TPU matrix were observed, particularly influencing the absorbance bands related to the -NH, C=O, and N-H groups. X-ray diffraction (XRD) analysis demonstrated enhanced crystallinity in the TPU nanocomposites, with new diffraction peaks and increased crystallite size correlating with higher jute CNF content. Field emission scanning electron microscopy (FESEM) revealed a uniform dispersion of the jute CNFs within the TPU matrix, contributing to improved interfacial adhesion and enhanced structural integrity. Thermal analysis using differential scanning calorimetry (DSC) and thermogravimetric analysis (TGA) showed an increase in the thermal stability, with the onset of degradation occurring at higher temperatures in the TPU/jute CNF nanocomposites. The glass transition temperature (T_g_) and melting temperature (T_m_) exhibited minor shifts, reflecting improved thermal performance. These findings suggest that the incorporation of jute CNFs significantly enhances the crystallinity, thermal stability, and structural organization of TPU, offering a sustainable approach for developing robust materials with potential applications in structural, corrosion-resistant, and high-performance fields.

## 1. Introduction

In recent decades, polymer materials and their composites have been widely utilized in various applications, including civil engineering [1], transportation [2], and medical treatment [3]. With advancements in science and technology, new polymer materials with enhanced mechanical, physical, and thermal properties have gained significant attention, leading to the development of polymer nanocomposites. Traditionally, inorganic fibers, such as glass and carbon, have been used as reinforcements in polymer nanocomposites. However, these synthetic fibers have major drawbacks, including limited environmentally friendliness [4,5] and high cost. To address these issues and develop greener materials, natural fibers are gaining interest as potential replacements for synthetic ones.

Natural fibers, such as jute, flax, hemp, kenaf, and palm, offer several advantages over synthetic fibers, including a lower density, easier processing, minimal health hazards, greater flexibility, and lower cost [5,6]. These sustainable natural fiber reinforcements have garnered significant interest in various industries due to their lower environmental impact [7], including reduced carbon emissions, lower fossil fuel consumption, and ease of fabrication, leading to the production of cost-effective and lightweight composite products [7,8]. As a result, natural fibers hold great potential as environmentally friendly materials that can substitute synthetic reinforcements, like glass and carbon fibers. Polymer matrix composites reinforced with natural fibers exhibit excellent mechanical properties and corrosion resistance, as fiber reinforcement serves as the primary load-bearing element, while the polymer matrix maintains the fiber orientation and position [9].

Among natural fibers, jute is considered the most cost-effective option due to its high tensile strength and affordability, being the second most produced natural fiber after cotton [10]. Jute fibers are at least 50% cheaper than other natural fibers, such as flax [11], making them particularly attractive for industrial applications. Their compatibility with polymers and favorable strength properties make them desirable for fabricating polymer composites used in applications such as affordable housing, grain storage, and small fishing boats [12]. Additionally, jute fibers have a lower content of non-cellulosic components compared to other natural fibers [6,13], which makes the surface modification process more effective in enhancing the adhesion between fibers and polymer matrices, resulting in improved mechanical and interfacial properties.

Recent studies have emphasized the potential of natural fibers, such as jute, as sustainable reinforcement materials in polymer matrices. One study found that incorporating jute CNFs into a poly (lactic acid) matrix significantly improved the tensile strength and thermal stability of nanocomposites [14]. Similarly, another study reported that cellulose nanofibers extracted from jute enhanced the mechanical properties of epoxy composites [15], while a separate study demonstrated improved tensile strength and modulus in TPU nanocomposites reinforced with cellulose nanocrystals [16]. Additionally, superior thermal stability and reduced water absorption were observed in TPU/cellulose nanofiber composites compared to the initial TPU [17].

The crystallinity and the presence of graphene reinforcement added to PU nanocomposites were studied using X-ray diffraction (XRD) analysis [18]. The highest intensity peak was observed at 2θ = 26.0° in the pure graphene spectrum, which was used as a reference. Upon incorporating graphene into the PU matrix, they identified the presence of graphene at a peak of 2θ = 26.0°. This shift in the diffraction angle from 2θ = 26.7° to 2θ = 26.0° confirmed the formation of two distinct crystalline structures, indicating the successful formation of the composite. In another study, researchers examined the effect of incorporating sugar palm fiber on the properties of TPU composites [19]. XRD analysis was conducted to examine the impact of different fiber sizes in the reinforced TPU composites, and no diffraction peak was observed in the TPU composite diffraction pattern. This absence of a peak suggests the complete exfoliation of sugar palm fibers within the TPU network structure. Additionally, a peak observed at 2θ = 23° indicates the amorphous nature of the composites, confirming the uniform dispersion of the sugar palm fiber within the TPU matrix and the compatibility between the reinforcement and matrix materials.

The interactions between leather particles and the TPU matrix during the fabrication of composites were investigated, and it was found that no chemical reaction occurred [20]. The FTIR spectrum showed stretching vibrations of hydrogen-bonded and free –NH groups at 3329 cm⁻^1^ and 3450 cm⁻^1^. Additionally, the amide I band (C=O), H-bonded (C=O), C-N stretching, and N-H bending (amide II band) were present in the spectrum of the TPU/leather particle composites. The absence of new peaks in the FTIR spectra confirmed that no chemical reaction occurred between the leather particles and the TPU matrix. Similarly, another study reported no significant chemical interactions between TPU and starch nanofiber reinforcements during processing [21]. FTIR analysis was used to study the polymer interactions and confirm whether any unfavorable reactions occurred between the TPU matrix and starch nanofiber reinforcement materials. The FTIR spectra of starch–TPU nanofiber-based composites were consistent with the findings [20], showing no significant peak changes and indicating that no unwanted chemical reactions occurred between the polymers during processing.

Integrating renewable jute CNFs into TPU nanocomposites represents a significant step forward in developing environmentally sustainable materials. At the end of their lifecycle, TPU/jute CNF composites are expected to exhibit greater biodegradability than traditional polymer composites, as natural fibers like jute are more readily broken down by microbial and enzymatic processes in natural environments. This degradation process is anticipated to produce non-toxic by-products—primarily carbon dioxide, water, and biomass—that help minimize ecological impact. The renewable nature of jute fibers, paired with the environmentally benign decomposition of these composites, underscores their potential to replace synthetic fiber-reinforced composites in applications requiring sustainable materials, contributing to waste reduction and supporting a circular economy in material science.

This study adds substantial value to the field of sustainable polymer nanocomposites by examining the incorporation of jute CNFs into TPU through melt blending. By varying the jute CNF content (2 wt% and 4 wt%), this research investigates the effects of nanofiber reinforcement on TPU structural and chemical properties using advanced analytical methods, including X-ray diffraction (XRD) and Fourier transform infrared (FTIR) spectroscopy. These analyses provide insight into how jute CNFs influence TPU phases and chemical properties, supporting the development of stable, homogeneous TPU composites with enhanced mechanical and interfacial characteristics.

Expanding on previous studies of PU/CNF composites, this work takes a unique approach by leveraging the nanoscale reinforcement of jute CNFs, offering improved compatibility with the TPU matrix. The cost-effectiveness and eco-friendliness of jute fibers further underscore the sustainability of this composite, positioning jute CNFs as a viable alternative to synthetic fibers. This focus on renewable, green materials aligns the research with global initiatives for environmentally sustainable materials and resource-efficient manufacturing.

In summary, this research advances the understanding of natural fiber-reinforced TPU composites and emphasizes the promise of jute CNFs as an eco-friendly, high-performance reinforcement option for TPU matrices. By advocating for a renewable, environmentally friendly approach to polymer reinforcement, this study sets the stage for future innovations in green nanocomposite materials.

## 2. Materials and Methods

**Figure 1:**
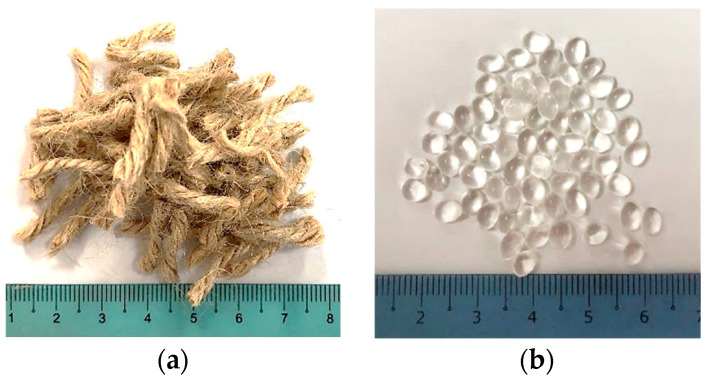
(**a**) Cut jute rope hemp; (**b**) granular TPU.

Thermoplastic polyurethane (TPU) and jute cellulose nanofibers (CNFs) were used as the matrix and reinforcement materials in this study. The TPU was provided in granular form by Mecha Solve Engineering Sdn. Bhd., while the jute fiber was supplied as hemp rope by Zheijang Hailun ROPE and Net Co., Ltd. (Taizhou, China), as shown in Figure 1a,b.

### 2.1. Preparation of CNFs from Jute Fiber

CNFs were extracted from the jute fibers using a chemo-mechanical method, combining both chemical and mechanical treatments. The process began by chemically treating the jute fibers to remove the non-cellulosic components, such as wax, lignin, hemicellulose, and other impurities. The jute fibers were first cut into 2–3 cm lengths and subjected to a swelling step, where they were immersed in an 18% *w/v* sodium hydroxide (NaOH) solution for 2 h to break down their structures. After swelling, the fibers were rinsed thoroughly with distilled water to neutralize the NaOH and then air-dried. Next, the fibers underwent hydrolysis in a 2M sulfuric acid (H_2_SO_4_) solution at 80 °C with continuous stirring for 3 h. After hydrolysis, the fibers were rinsed with excess distilled water until the pH was neutral and air-dried. To remove soluble lignin, the fibers were immersed in a 3% *w/v* NaOH solution at 80 °C for 3 h, again with continuous stirring, followed by another round of rinsing and air-drying. Finally, the fibers underwent a bleaching process in 2% *v/v* sodium chlorite (NaClO_2_) solution at 50 °C for 1 h. This bleaching process was repeated 2 to 3 times until the residue became colorless. The treated fibers were cooled, filtered, rinsed, and dried. After the chemical treatment, the fibers were mechanically treated through pulverization, carried out under wet conditions using a high-energy planetary mill (Retsch-PM 100, Retsch GmbH, Haan, Germany) at Univeristi Teknikal Malaysia (UTEM). Deionized water was used in an agate jar with zirconia balls (10 mm diameter) in a 10:1 ratio, operating at 450 rpm for 6 h at room temperature. The resulting slurry was then freeze-dried at −50 °C for 8 h.

### 2.2. Preparation of TPU Nanocomposite

Two different compositions (2% and 4% by weight) of TPU/jute CNF nanocomposites were prepared using a melt blending technique. The TPU granules were preheated at 80 °C for 6 h in an oven to eliminate the moisture content. The TPU and jute CNFs were then mixed and compounded in an internal mixer (ThermoHAAKE Polylab Rheomix, Thermo Fisher Scientific, Waltham, MA, USA) at 180 °C, with a rotor speed of 150 rpm. The blended materials, containing different weight percentages (2% and 4%) of jute CNFs, were subjected to hot compression into 150 × 150 mm^2^ square sheets with a 3 mm thickness mold at 180 °C for 10 min under 5 MPa compression pressure. The resulting sheets were cooled to room temperature under pressure and then cut into cuboid-shaped specimens (1 × 1 × 0.3 cm^3^) using a die cutter, following the ASTM D638-14 standards [22] for XRD and FTIR analysis. These processes were conducted at Universiti Tun Hussein Onn Malaysia (UTHM).

### 2.3. Fourier Transform Infrared (FTIR) Spectroscopy

FTIR analysis was performed to investigate the organic composition and chemical characteristics of both the initial TPU and TPU/jute CNF nanocomposites. This technique was used to identify structural changes in the TPU following the incorporation of jute CNFs. Since the TPU/jute CNFs were in sheet form, the attenuated total reflectance (ATR) method was used, with the nanocomposite sheet placed directly onto the ATR crystal. The infrared spectra of the initial TPU and TPU/jute CNF nanocomposites were recorded using a Thermo Scientific FTIR spectrometer, covering wavenumbers from 800 to 4000 cm^−1^ to observe the absorbance or transmittance of infrared light, generating the spectra.

### 2.4. X-Ray Diffraction (XRD)

XRD analysis was conducted to assess the phase composition and crystallinity of both the initial TPU and TPU/jute CNF nanocomposites. The XRD patterns of the samples were obtained using a Panalytical x’pert Pro instrument, employing CuKα radiation (λ = 0.15406 nm) from a copper (Cu) anode. Scans covered a 2θ range from 10° to 90° with a scanning rate of 2°/min.

## 3. Results and Discussion

### 3.1. Phase Analysis of Initial TPU and TPU/Jute CNF Nanocomposites by XRD

**Figure 2:**
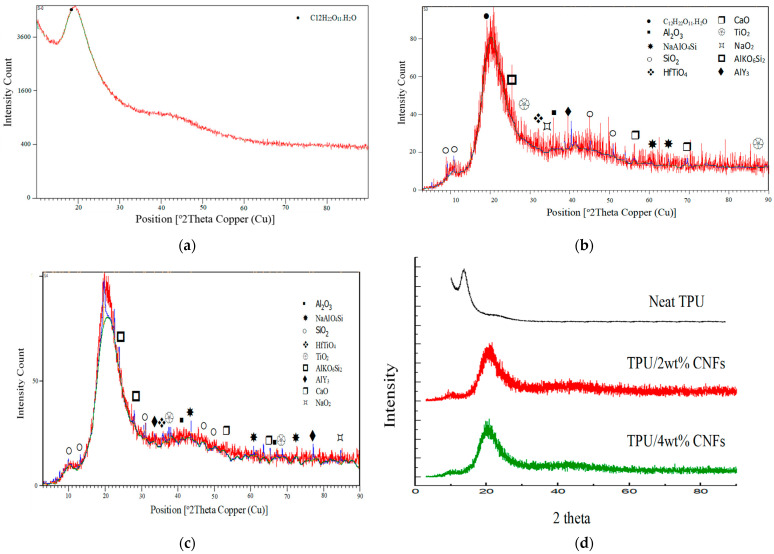
XRD spectra of (**a**) initial TPU (**b**) TPU/2 wt% jute CNF nanocomposites; (**c**) TPU/4 wt% jute CNF nanocomposites; (**d**) combined initial TPU, TPU/2 wt%, and 4% jute CNF nanocomposites.

The XRD spectra of the initial TPU, TPU with 2 wt%, and TPU with 4 wt% jute CNF nanocomposites are shown in Figure 2a–c, respectively. The XRD pattern of the initial TPU displays a broad diffraction peak centered at 2θ = 19.35° (intensity count = 5173) with diffuse peaks, indicating the amorphous nature of TPU. However, the presence of organized crystalline regions, characterized by intense peaks, corresponds to the microphase-separated structure of TPU, where hard segments are crystalline and soft segments are more amorphous. Additionally, the compound hydroxymethyl (C_12_H_22_O_11_.H_2_O) (ICDD 00-027-1947) was observed at 2θ = 18.36°, representing the hard segments of the TPU matrix. Here, the hard segment content of 50 mass% was modified by the presence of a carboxylic group through hydroxymethyl, a finding also reported in [23]. After the addition of 2 wt% jute CNFs, notable features in the XRD pattern (Figure 2b) include a reduction in the intensity count and enhanced TPU ordering, evidenced by the appearance of ten peaks at 2θ = 9.05°, 21.39°, 28.87°, 30.58°, 35.86°, 46.19°, 61.05°, 54.03°, 57.67°, 77.39°, and 81.46°, which signify a more crystalline order. These peaks represent trace elements and compounds present in the jute CNFs. Due to the low loading of jute CNFs, they are embedded within the TPU matrix, resulting in a peak for jute CNFs (200) at 2θ = 9.11°. Moreover, the broad diffraction peak of TPU shifted from 2θ = 19.35° to 21.39°, indicating the presence of cellulose II introduced by the jute CNFs. As the jute CNF content increased to 4 wt%, additional notable features included an increase in intensity and the emergence of peaks appearing at 2θ = 9.11°, 24.01°, 28.87°, 30.58°, 35.86°, 60.71°, 63.35°, 75.63°, and 86.84°. These peaks indicate an increasing presence of elements and compounds from the jute CNFs embedded in the nanocomposites, along with further intercalation processes. The XRD analysis of jute CNFs was previously discussed in a study on XRD and FTIR analyses of extracted jute CNFs [13].

CNFs act as nucleating agents, promoting the crystallization of TPU. This is evident in the XRD results, which demonstrate increased crystallinity with the addition of CNFs. The enhanced crystalline structure contributes to improved mechanical properties [3], as it enables a more uniform stress distribution across the composite, thereby reducing the likelihood of crack propagation and material failure.

Furthermore, crystallite size can be calculated from XRD analysis using the Debye–Scherrer equation. In this study, the crystallite size of the fabricated TPU/jute CNF nanocomposites was determined, revealing that the incorporation of jute CNFs led to increased crystallinity. Specifically, the crystallite size grew from 0.67 nm for 2 wt% CNFs to 0.73 nm for 4 wt% CNFs, highlighting the structural enhancement induced by the nanofibers. Similar findings were observed on the TPU synthesis with modified organophilic montmorillonite (OMMT) [24].

Additionally, the differing Y-axis scales in the XRD patterns of the initial TPU and the TPU nanocomposites reflect the varying concentrations of TPU and CNFs in each sample. The scale for the initial TPU (Figure 2a) indicates higher absorbance due to the bulk material, while the scales for TPU with 2 wt% and 4 wt% CNFs (Figure 2b,c) show lower absorbance due to the increased proportion of CNFs. Normalizing the spectra for these variations is essential for accurate comparison and interpretation.

### 3.2. FTIR Analysis of Initial TPU and TPU/Jute CNF Nanocomposites

**Figure 3:**
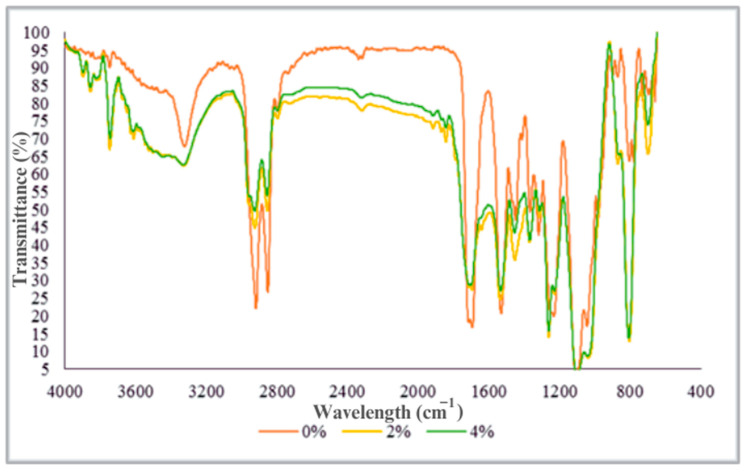
FTIR spectra of initial TPU, 2 wt% jute, and 4 wt% jute CNF nanocomposite.

FTIR analysis was conducted on the initial TPU and TPU/jute CNF nanocomposites to investigate interfacial interactions within the TPU nanocomposites and to identify any adverse interactions that might occur during processing (Figure 3).

Figure 3 presents the FTIR spectra of both the initial TPU and TPU/jute CNF nanocomposites, spanning from 600 to 4000 cm^−1^. Notably, the incorporation of jute CNFs induced significant spectral changes across the single bond, triple bond, double bond, and fingerprint regions. In the 3600–4000 cm^−1^ region, a distinct absorption band corresponding to O-H stretching vibrations is observed in the initial TPU. This band intensifies with the addition of 2 wt% and 4 wt% jute CNFs, highlighting the increased presence of non-bonded hydroxyl groups. A broad absorption band between 3200 and 3600 cm⁻^1^, associated with hydrogen bonds, further confirms hydrate (H₂O) or hydroxyl (O-H) stretching vibrations. The increased band width with 2 wt% and 4 wt% CNFs reflects the impact of O-H groups in the jute CNFs. In the 2800–3200 cm⁻^1^ region, the TPU spectra show strong doublet bands indicative of alkyl chains (e.g., polyalcohols, saccharides, and fats) involved in C-H stretching vibrations, consistent with the findings in [25]. With the inclusion of 2 wt% and 4 wt% jute CNFs, these bands decrease in intensity—by 20% and 30%, respectively—suggesting that the jute CNFs influence the dipole moment of the C-H bond.

A minor but notable change occurs in the triple bond region (2000–2500 cm⁻^1^), where a weak C≡C absorbance band (medial alkyne stretching vibration) diminishes and nearly disappears with increasing CNF content. Concurrently, a new absorption band emerges in the double bond region (1500–2000 cm⁻^1^), indicating C=O stretching vibrations possibly from active carbonyl groups, such as anhydrides or organic carbonates (e.g., CaCO_3_), as confirmed by XRD and XRF analyses of the jute CNFs [3,13]. Additionally, two prominent absorbance bands in the double bond region (1530 cm⁻^1^ and 1690–1715 cm⁻^1^) in the initial TPU, associated with aromatic nitro and carboxylic or ketone stretching, also show decreased intensity with CNF incorporation.

In the fingerprint region (1500–400 cm⁻^1^), several sharp and weaker duplex bands in the initial TPU spectra indicate compounds, like common inorganic ions (e.g., carbonate and nitrate), phosphorus–oxygen compounds (e.g., aromatic phosphates), organic silicon (Si₂O), aliphatic chloro compounds (C-Cl stretching), and aromatic rings. The addition of 2 wt% and 4 wt% jute CNFs reduces the absorbance intensity in this region, leading to a higher transmittance percentage. This trend suggests reduced frequency absorption, influenced by factors such as changes in the dipole moment, bond quantity, and particle size, likely due to the nanoscale structure of the jute CNFs [26]. Both TPU and cellulose possess functional groups capable of forming hydrogen bonds. TPU includes urethane linkages with N-H and C=O groups, while cellulose is rich in hydroxyl (-OH) groups. These hydrogen bonds foster strong interfacial adhesion between CNFs and the TPU matrix, facilitating stress transfer and thereby enhancing the composite’s tensile strength and modulus [3].

### 3.3. Field Emission Scanning Electron Microscopy (FESEM) Analysis of Initial TPU and TPU/Jute CNF Nanocomposites

**Figure 4:**
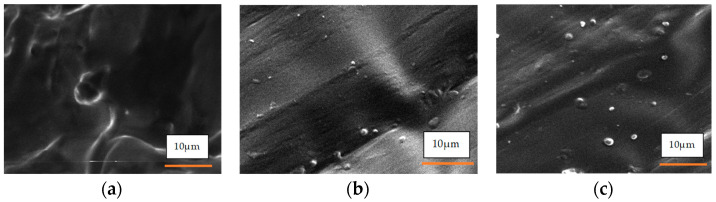
FESEM of (**a**) initial TPU; (**b**) TPU/2 wt% jute CNF nanocomposite; (**c**) TPU/4 wt% jute CNF nanocomposite.

The FESEM images (Figure 4a–c) provide clear visual evidence of the morphological changes between the initial TPU and the TPU reinforced with 2 and 4 wt% jute CNFs. The surface of the initial TPU appears relatively smooth and homogeneous, reflecting its amorphous nature, as confirmed by XRD analysis, which shows no distinct crystalline peaks. This smooth surface, with only minor irregularities and particles visible, likely arises from TPU’s inherent surface roughness in the absence of reinforcing agents. In contrast, the surfaces of TPU with 2 and 4 wt% jute CNFs display significant texturing and roughness, with visible fiber-like structures embedded within the matrix. These observations confirm the successful incorporation and dispersion of jute CNFs, resulting in a more structured morphology. The increased surface roughness and presence of CNFs align with the enhanced crystallinity observed in the XRD analysis and suggest stronger interfacial bonding between the TPU matrix and the CNFs, as evidenced by the FTIR analysis. This improved bonding, combined with the enhanced surface morphology, indicates that the nanofibers are well integrated into the TPU matrix, likely contributing to the improved thermal properties. Overall, the structural differences between the initial TPU and TPU/jute CNF nanocomposites underscore that the addition of jute CNFs resulted in significant phase and property modifications in the TPU matrix.

**Figure 5:**
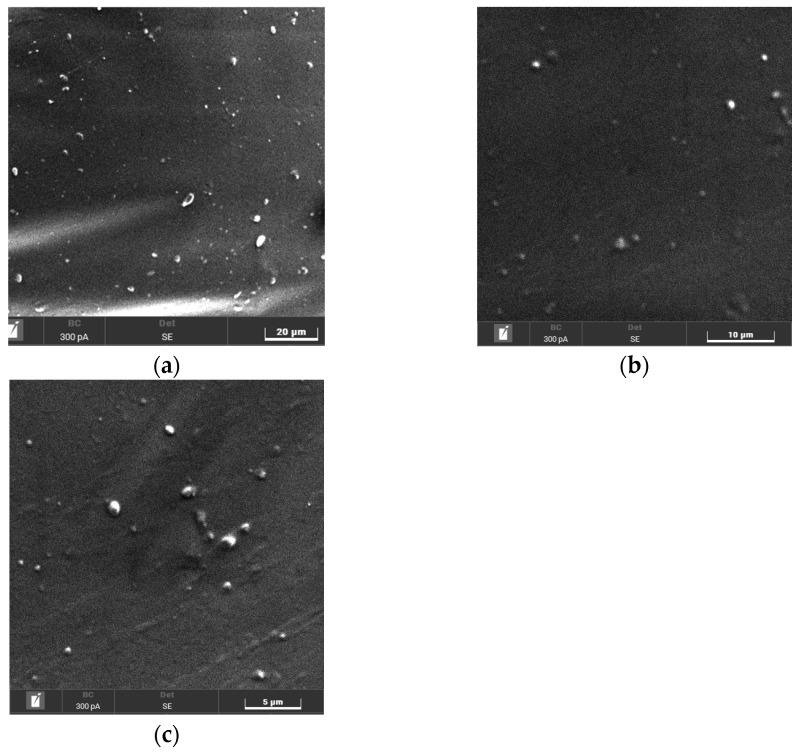
FESEM of 4 wt% jute CNF nanocomposite at different magnifications: (**a**) 1000×; (**b**) 3k×; (**c**) 5k×.

The FESEM images in Figure 5, captured at magnifications of 1k×, 3k×, and 5k× for the 4 wt% jute CNF nanocomposite, provide detailed insights into the interfacial morphology and distribution of CNFs within the TPU matrix. At the lower magnification (1k×), a more extensive view of the nanocomposite surface reveals an uneven texture, indicating the incorporation of jute CNFs, which disrupts the otherwise smooth surface characteristic of pure TPU, as previously seen in Figure 4. As the magnification increases to 3k× and 5 k×, the images show finer details of the nanofiber distribution, with visible white dots likely representing embedded jute CNFs. The roughened morphology and presence of CNFs suggest effective dispersion within the TPU, contributing to its enhanced crystallinity and structural integrity, consistent with the XRD results. This textured interface, along with the interfacial bonding observed in the FTIR analysis, indicates a well-integrated composite structure, enhancing the thermal stability of the material. These observations confirm that the jute CNFs were successfully incorporated and dispersed, leading to a modified TPU structure and improved nanocomposite performance.

### 3.4. Thermal Analysis

#### 3.4.1. Differential Scanning Calorimetry (DSC) Analysis of Initial TPU and TPU/Jute CNF Nanocomposites

**Figure 6:**
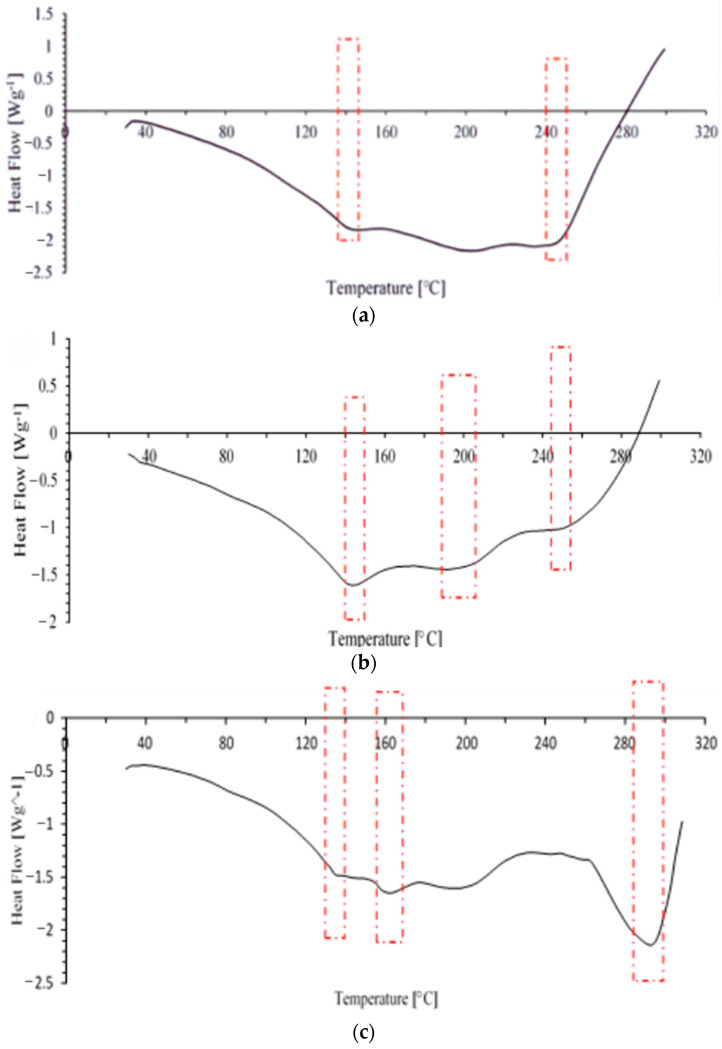
DSC analysis, temperature versus heat flow of (**a**) initial TPU; (**b**) TPU/2 wt% jute CNF nanocomposite; (**c**) TPU/4 wt% jute CNF nanocomposite.

The thermal analysis of the initial TPU and the TPU with 2 and 4 wt% jute CNFs was investigated using differential scanning calorimetry (DSC) to examine the thermal transitions, as illustrated in Figure 6a–c.

For the initial TPU (Figure 5a), the DSC results reveal distinct thermal transitions. The first major endothermic peak appears between 110 °C and 150 °C, corresponding to the glass transition (T_g_) of TPU. This decrease in the heat flow suggests a phase change, with TPU transitioning from a glassy to a more rubber-like state. Following this, a second endothermic peak occurs at around 240 °C, associated with the onset of melting within TPU’s crystalline domains, indicating thermal softening as these regions begin to break down. An exothermic peak near 280 °C marks the start of TPU thermal degradation.

In the TPU with 2 wt% jute CNFs (Figure 5b), the DSC profile shows three endothermic peaks at 143.3 °C, 191.5 °C, and 251 °C. The first peak, between 140 °C and 160 °C, represents the melting of a slow crystallizing phase, while the peak at 191 °C reflects the melting of the fast crystallization phase in the TPU matrix. The endothermic peak around 251 °C corresponds to the reaction temperature of cellulose fibers, typically occurring between 200 °C and 300 °C. This characteristic of cellulose fibers results in an increase in the melting temperature of TPU nanocomposites with added jute CNFs, as anticipated.

The DSC profile for the TPU with 4 wt% jute CNFs (Figure 5c) shows a similar pattern to the initial TPU, although there are shifts in the peak temperatures and overall heat flow. The Tg remains within the 110 °C to 150 °C range but with a less pronounced endothermic peak, indicating reduced chain mobility in the polymer due to the incorporation of jute CNFs. This restriction on polymer chain movement reduces heat absorption during the transition, while the slight increase in the melting temperature (T_m_) suggests improved thermal stability. Although the exothermic peak for thermal degradation occurs at a somewhat lower temperature than in the initial TPU, the overall thermal resistance improves with jute CNF reinforcement.

The inclusion of jute CNFs in the TPU matrix notably altered its thermal behavior, especially in the soft segment melting and hard segment degradation regions. The nanocomposites exhibited enhanced thermal stability, reflected in the higher temperature range for the hard segment transition. This enhancement likely arose from strong interactions between the jute CNFs and the TPU matrix, which restricted the polymer chain mobility and improved the resistance to thermal degradation. The reduced intensity of the thermal transitions suggests that the jute CNFs also influenced the heat absorption, contributing to a more stable thermal profile compared to the initial TPU.

#### 3.4.2. Thermogravimetric Analysis of Initial TPU and TPU/Jute CNF Nanocomposites

**Figure 7:**
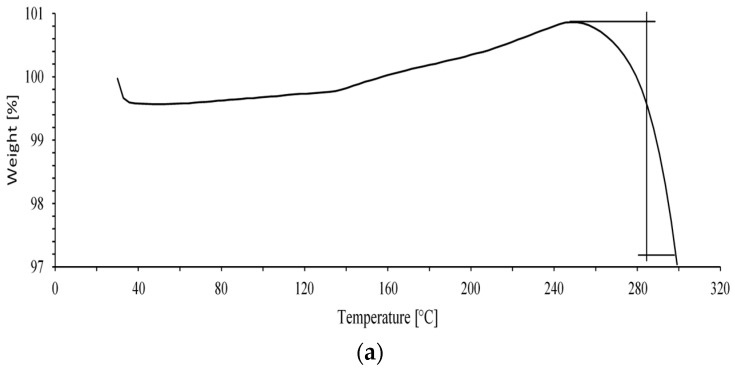

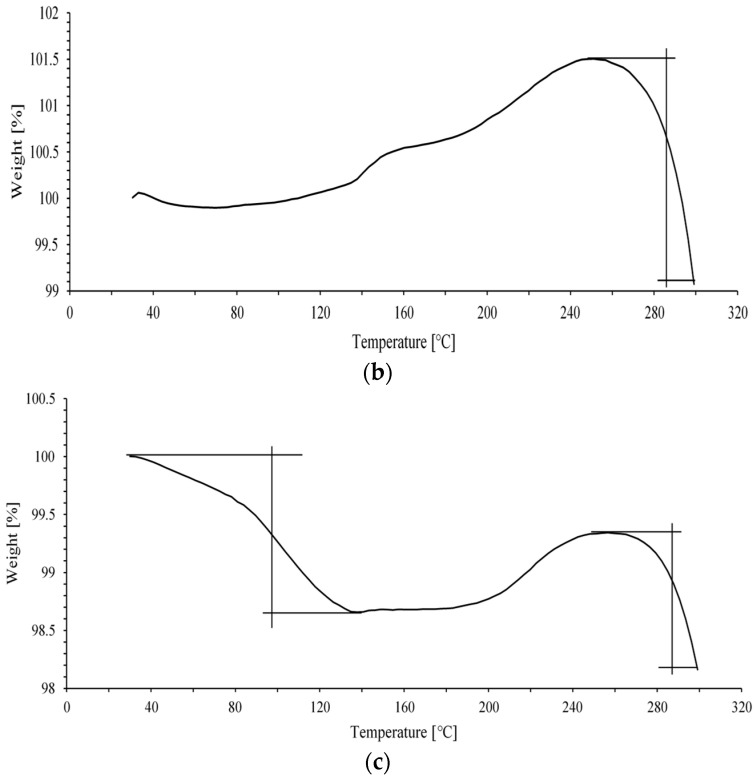
TGA, temperature versus wt.% of (**a**) initial TPU; (**b**) TPU/2 wt% jute CNF nanocomposite; (**c**) TPU/4 wt% jute CNF nanocomposite.

The thermal analysis of the initial TPU and the TPU reinforced with 2 and 4 wt% jute CNFs provides valuable insights into the thermal stability and degradation behavior of these materials. Figure 7a–c illustrate the thermogravimetric analysis (TGA) results for the initial TPU, TPU with 2 wt% jute CNFs, and TPU with 4 wt% jute CNFs, respectively.

The TGA curve of the initial TPU (Figure 7a) shows a smooth and gradual weight loss as the temperature increases. The material starts to degrade at around 230 °C, with its most significant weight loss occurring at approximately 280°C, where the weight percentage drops sharply. The single-step degradation pattern is characteristic of TPU, indicating a straightforward thermal breakdown within a narrow temperature range. The absence of any reinforcement results in limited thermal stability, as no secondary phases or reinforcement agents are present to resist thermal degradation.

With the addition of 2 wt% jute CNFs (Figure 7b), the degradation profile shows a minor change, with an additional peak at around 160 °C. This peak likely corresponds to the initial stages of the thermal degradation of cellulose, a primary component of the jute CNFs, which typically decomposes between 150 °C and 200 °C. The exothermic peak at 160 °C may indicate the breakdown of cellulose into volatile compounds, often accompanied by a release of heat. This early degradation behavior suggests an interaction between the TPU matrix and the jute CNFs, with the cellulose contributing to the thermal profile of the composite.

The thermal degradation of the TPU with 4 wt% jute CNFs (Figure 7c) exhibits a distinct multi-step degradation profile. Although the initial onset of weight loss occurred slightly earlier than in the initial TPU, the overall thermal stability was improved due to multiple stages of weight loss. The initial weight loss, observed between 80 °C and 150 °C, is likely associated with the evaporation of moisture or volatile components. A notable degradation peak appears at around 280 °C, similar to the initial TPU, though the weight loss occurred more gradually, reflecting enhanced thermal stability.

The multi-step degradation observed in the TPU/jute CNF nanocomposite suggests that the nanofibers influenced the decomposition process, likely due to their strong interfacial interaction with the TPU matrix. The inclusion of jute CNFs improved the thermal stability by restricting the thermal motion of the polymer chains, thereby delaying the material’s thermal decomposition. This enhanced thermal performance was further supported by the increased crystallinity shown in the XRD analysis and the strong interfacial bonding confirmed by FTIR, indicating that jute CNFs serve as an effective reinforcement agent within the TPU matrix.

## 4. Conclusions

This study successfully demonstrated the enhancement of thermoplastic polyurethane (TPU) properties through the incorporation of jute cellulose nanofibers (CNFs). The addition of jute CNFs significantly improved the crystalline structure and chemical characteristics of TPU, as evidenced by X-ray diffraction (XRD) and Fourier transform infrared (FTIR) analyses. Specifically, XRD analysis confirmed the successful integration of jute CNFs within the TPU matrix, showing a reduction in the amorphous regions of TPU and an increase in crystallite size with the addition of 2 wt% and 4 wt% jute CNFs. FTIR spectroscopy further indicated that the inclusion of nanosized jute CNFs altered the intensity and frequency of the absorbance bands, particularly those associated with the NH, C=O, and N-H groups in TPU, suggesting strong interfacial interactions between the CNFs and TPU’s functional groups. Field emission scanning electron microscopy (FESEM) revealed a uniform dispersion of jute CNFs within the TPU matrix, which contributed to improved interfacial adhesion and a more organized nanostructure. This improved nanostructure played a significant role in enhancing the structural and thermal properties of the TPU/jute CNF nanocomposites. Thermal analysis showed that the incorporation of jute CNFs resulted in enhanced thermal stability, with the TPU nanocomposites exhibiting a higher onset degradation temperature and improved thermal performance. Minor shifts in the glass transition and melting temperatures were observed, indicating that the jute CNFs contributed to reinforcing the TPU matrix without compromising its thermal characteristics.

The incorporation of jute CNFs into TPU has proven to be an effective strategy for enhancing crystallinity, thermal stability, and interfacial adhesion, which are crucial factors in advancing the performance of polymer nanocomposites. This study contributes to the broader field of sustainable nanocomposite materials by offering a viable approach to improve material properties through natural fiber reinforcement, underscoring the significance of CNFs as eco-friendly, performance-enhancing additives.

The unique combination of enhanced crystallinity, thermal stability, and interfacial adhesion in TPU/jute CNF nanocomposites highlights their suitability for a range of applications requiring robust, sustainable materials. In the automotive industry, the improved thermal stability and structural integrity make these nanocomposites ideal for high-performance interior parts and lightweight components that must withstand temperature variations while ensuring durability and safety. For aerospace applications, the high crystallinity and strong interfacial adhesion of TPU/jute CNF nanocomposites enable the fabrication of lightweight structural parts with superior mechanical strength and stability, supporting greater fuel efficiency and material longevity. Similarly, in the construction sector, the thermal stability and enhanced structure of TPU/jute CNF nanocomposites meet the demand for sustainable materials capable of providing insulation and withstanding environmental stresses. These nanocomposites show promise for use in building insulation, protective coatings, and lightweight structural materials, offering a viable alternative to traditional materials.

Future research should explore the scalability of the production process, assess the long-term stability and performance of these nanocomposites in diverse environmental conditions, and evaluate the biodegradability of the composites to support sustainable and eco-friendly applications. Additionally, functionalizing the jute CNFs to introduce properties, such as antimicrobial activity or enhanced electrical conductivity, could expand their application potential. With continued research and development, TPU/jute CNF nanocomposites could play a pivotal role in the development of sustainable, high-performance composite materials for a wide range of industrial applications.

## Data Availability

The original contributions presented in the study are included in the article, further inquiries can be directed to the corresponding author.
